# REP1 Modulates Autophagy and Macropinocytosis to Enhance Cancer Cell Survival

**DOI:** 10.3390/ijms18091866

**Published:** 2017-08-28

**Authors:** Jungwon Choi, Hyena Kim, Young Ki Bae, Heesun Cheong

**Affiliations:** Tumor Microenvironment Research Branch, Research Institute, National Cancer Center, 323 Ilsan-ro, Ilsandong-gu,10 Goyang-si, Gyeonggi-do 10408, Korea; 73801@ncc.re.kr (J.C.); 74490@ncc.re.kr (H.K.)

**Keywords:** rab escort protein 1 (REP1), lysosome, autophagy, macropinocytosis, mTORC1

## Abstract

Rab escort protein 1 (REP1), a component of the Rab geranyl-geranyltransferase 2 complex, plays a role in Rab protein recruitment in proper vesicles during vesicle trafficking. In addition to having well-known tissue degenerative phenotypes in the REP1 mutant, REP1 is tightly associated with cancer development and contributes to cell growth and survival. However, the functional mechanism of REP1 in cancer progression is largely uninvestigated. Here, we show that REP1 plays a crucial role in regulating mammalian target of rapamycin (mTOR) signaling and its downstream pathways, as well as autophagy and macropinocytosis, which are essential for cancer cell survival during metabolic stresses including starvation. REP1 small interfering RNA (siRNA) treatment downregulates mTORC1 activity in growing media, but blocks autophagosome formation under nutrient-depleted conditions. In contrast to the mild decrease of lysosomal enzyme activity seen in REP1 depletion, in REP1 knockdown the subcellular localization of lysosomes is altered, and localization of REP1 itself is modulated by intracellular nutrient levels and mTOR activity. Furthermore, REP1 depletion increases macro pinocytosis which may be a feedback mechanism to compensate autophagy inhibition. Concomitant treatment with macropinocytosis inhibitor and REP1siRNAresults in more significant cell death than autophagy blockade with REP1 knockdown. Therefore, REP1-mediated autophagy and lysosomal degradation processes act as novel regulatory mechanisms to support cancer cell survival, which can be further investigated as a potential cancer-targeting pathway.

## 1. Introduction

Autophagy is a lysosome-mediated degradation process in which unnecessary organelles and macromolecules are sequestered into autophagic vesicles and are delivered to lysosomes. Ultimately, various autophagic cargo molecules are degraded and recycled back to maintain cellular bioenergy homeostasis and support biosynthetic processes under metabolic stress conditions including nutrient deprivation [1,2]. Multiple growth-signaling pathways negatively regulate autophagy, a typical catabolic pathway. The serine/threonine protein kinase, mammalian target of rapamycin (mTOR) acts as a nutrient-sensing molecule to promote cell growth and suppress autophagy, whereas mTOR inactivation in response to nutrient deprivation induces autophagy [3,4]. Subsequent to autophagy activation in response to stresses, lysosomes play a pivotal role in degrading a variety of cargo molecules effectively, dependent on or independent of the autophagy pathway, using multiple trafficking routes. Moreover, lysosomes have recently received attention as a platform for regulating the mTORC1 pathway. Lysosomal function and localization can be markedly affected by mTORC1 activity. The subcellular localization of mTOR depends on intracellular nutrient status, particularly amino acid levels [5]. In nutrient-complete conditions, mTOR with its active state is located at the lysosomal membrane. In contrast, inactive mTOR is no longer localized at the lysosomal membrane during nutrient starvation, but is more diffusely distributed throughout the cytoplasm instead [6,7,8,9,10,11]

Rab escort protein 1 (REP1) is essential for lipid modification of Rab proteins, directly interacting with most Rab proteins and presenting them to Rab-geranyl-geranyltransferase (GGTase2). Subsequently GGTase2 transfers the geranyl-geranyl moiety to the target proteins such as RabGTPases. This lipid modification step, called “prenylation”, dictates that Rab proteins localize at the cell membrane and further maintain their active status in a GTP-bound form at the proper locations. Therefore, since RabGTPases are involved in various steps of vesicle trafficking, it is postulated that REP1 has critical role in vesicle trafficking via regulating RabGTPase activity [12,13].

As an aspect of pathophysiology, mutations in *REP1* cause a disease called choroideremia (CHM), which is known as an X-linked eye disease resulting from progressive degeneration of multiple tissues including photoreceptors, retinal pigment epithelium, and choriocapillaris [14,15].

In addition to defective phenotypes of eye-degeneration, REP1 mutant in zebrafish have also shown excessive cell death in various organs during embryonic development [16,17]. Moreover, REP1 is highly expressed in various tumors and has an oncogenic effect via preventing epidermal growth factor receptor (EGFR) degradation [18]. As another mechanism supporting for cell survival, REP1 directly interacts with fork head box O3 (Foxo3) to suppress nuclear localization of this transcription factor, which is associated with expression of the apoptotic gene set, demonstrating that REP1 prohibits cell death in response to multiple stress conditions including serum starvation or anticancer drug-5FU-treatment [19]. Overall results from previous reports indicate that REP1 plays an important role for cell growth and survival, particularly under metabolic stress conditions including nutrient deprivation [18,19].

In this study, we suggested that REP1 contributes to certain types of intracellular vesicle trafficking such as autophagy, which is activated by metabolic stress conditions. Autophagy and macro pinocytosis have critical roles in supporting cell growth and survival of cancer cells. Based on increasing sensitivity of REP1 deficiency in response to nutrient deprivation, we investigated the role ofREP1 in regulation of mTORC1 activity and autophagy. Moreover, we examined the importance ofREP1 in subcellular localization of lysosome using the cells transfected with REP1 small interfering RNA (siRNA). Meanwhile, we also examined the subcellular localization of REP1, which can be affected by mTOR activity. Overall findings indicate that REP1 plays a critical role in recruiting organelles and vesicles which are involved in the degradation processes at the proper place. Since pancreatic cancer cells maintain intracellular levels of nutrients via pathways of both autophagy and macropinocytosis, REP1-mediated autophagy regulation could be a potential therapeutic target for treating pancreatic cancer.

## 2. Results

### 2.1. REP1 Regulates Cell Growth and Survival in Pancreatic Cancer Cell Lines

**Figure 1:**
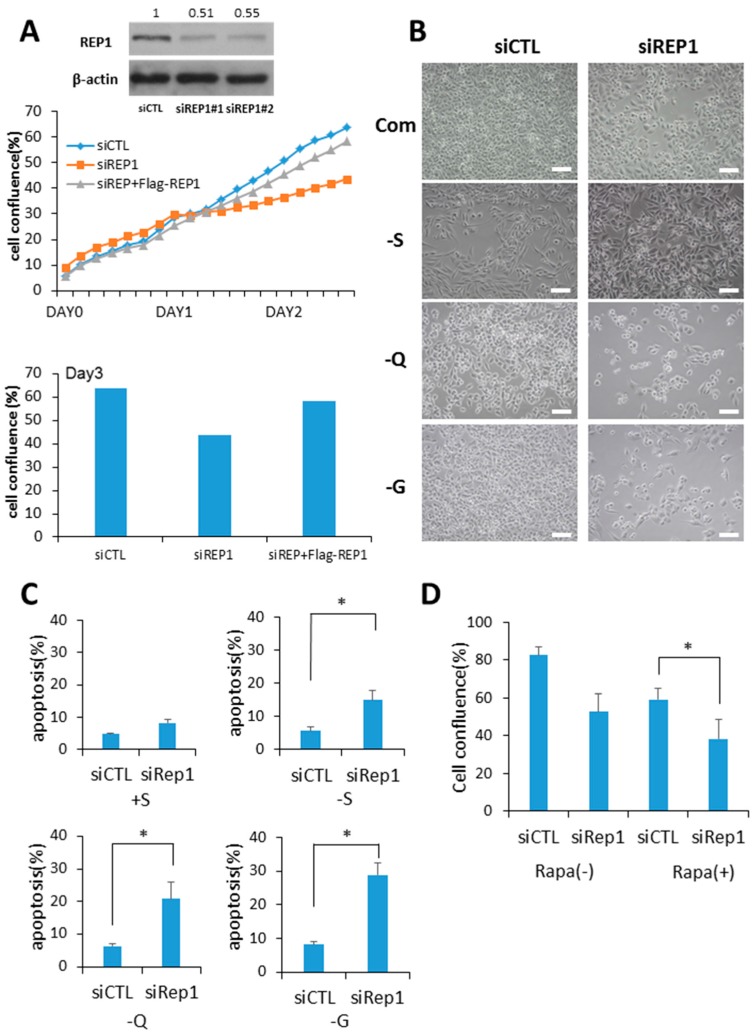
Rab escort protein1 (REP1) depletion suppresses cell growth and survival. (**A**) MiaPaCa2 cells were transfected with control (CTL) and REP1 small interfering RNAs (siRNAs). After 48 h, immunoblotting was performed to analyze REP1 protein levels. Cells were treated with CTL and REP1 siRNAs and incubated for 24 h. Then, the cells were transfected with Flag-REP1 plasmid additionally and further incubated in the IncuCyte^TM^ for monitoring cell proliferation. At the 72-h incubation time point, cell confluence levels were presented as a percentage using the IncuCyte^TM^ analyzer. (**B**,**C**) MiaPaCa2 cells were transfected with control and REP1siRNAs, which replaced the following day with serum-, glucose-, or glutamine-free medium and then incubated for another 24 h.Cell morphology was observed by brightfield image. Scale bar: 50 μm (**B**). Cell death was assessed by using the Annexin V/propidium iodide (PI) assay (**C**). Error bars indicate mean +/− standard error for *n* = 3 independent experiments. (**D**) MiaPaCa2 cells were transfected with control (CTL) or REP1siRNAs, which replaced the following day with 1 µM rapamycin and then further incubated for monitoring cell confluence using IncuCyte^TM^. At 72 h time point, cell confluence levels were presented as percentage. Statistical significance was determined via t-test; * *p* < 0.05.

Since a predominant function of REP1 is prenylation of Rab proteins via recruitment with active Rab geranylgeranyl transferase (RabGGTase), we hypothesized that REP1 might be critical for intracellular vesicle trafficking, closely related to growth signaling pathways. To explore the functional effect of REP1 in cancer cells, cells of the pancreatic cancer cell lines MiaPaCa2 and 8988T were transfected with REP1 small interfering RNA to knock down REP1.Compared to treatment with control siRNA, two independent REP1siRNA transfections reduced protein levels of REP1, as determined by immunoblot analysis (Figure 1A).

Next, we investigated whether REP1 knockdown in cancer cells influences cell growth and examined whether deprivation of glucose or glutamine, known as two major energy sources for cancer growth, influences cell growth and survival. REP1 knockdown with siRNA treatment results in a significant cell growth delay in a nutrient-complete medium using a pancreatic cancer cell line, which was almost rescued when REP1 was overexpressed (Figure 1A and Figure S1). Consistent with results from recent reports [18,19], REP1 knockdown in cancer cells results in a severe growth defect under nutrient deprivation when we observed these cells at 48 h after nutrient withdrawal with limited levels of either glucose or glutamine (Figure 1B). To investigate the effect of REP1 on cell survival under nutrient-deprived conditions, we performed a cell death assay using Annexin V/propidium iodide (PI) apoptosis analysis with suppression of REP1 in treatment with siRNA. REP1 knockdown in the cells substantially increased the populations of Annexin V-stained apoptotic cells in response to either glucose or glutamine deprivation conditions (Figure 1C), indicating that REP1 depletion sensitizes cancer cells, particularly in association with the metabolic stress derived from nutrient deprivation. Accordingly, next we examined whether cell growth suppression by REP1 knockdown can be affected by modulation of mTOR activity, a typical nutrient signaling pathway. Indeed, cell growth was more significantly decreased in REP1 knockdown cells with rapamycin treatment compared with either suppression of REP1 or mTORC1 inhibition with rapamycin alone (Figure 1D). These data imply that REP1 might have a distinct role in cell growth and survival, particularly in response to nutrient deprivation, by regulating the nutrient-signaling pathway.

### 2.2. REP1 Plays a Role in Regulating the mTORC1 Signaling Pathway

**Figure 2:**
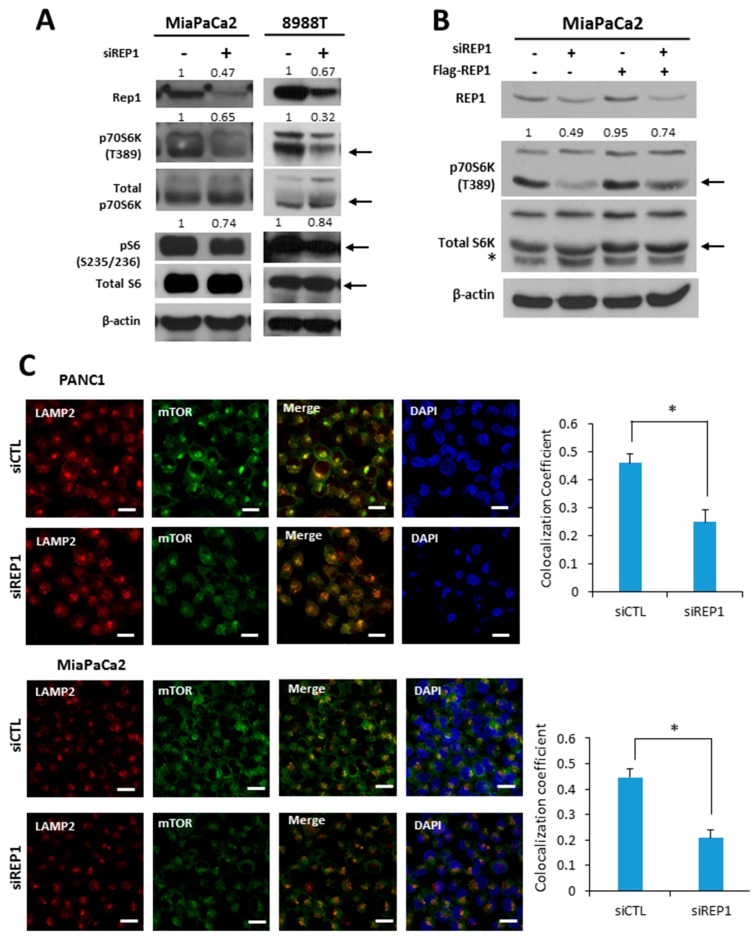
REP1 knockdown down-regulates mTORC1 signaling pathway. (**A**) Cells were treated with CTL orREP1siRNAs for 48 h. Then, cells harvested and the lysates were immunoblotted with antibodies against REP1, p70S6K, pS6, total 70S6K, S6 ribosomal (S6) protein, and beta actin; (**B**) MiaPaCa2 cells were with CTL or REP1siRN as for 24 h. Then, Flag-REP1 plasmid were transfected to the cells for overexpression, which were harvested and immunoblotted with antibodies against p70S6Kand a total 70S6K. These results are representative of three independent experiments; (**C**) cells were treated with CTL orREP1siRNAs for 48 h. After 2 h incubation in the presence or absence of amino acids, cells were stained with antibodies against mTOR and LAMP2, and analyzed by fluorescence microscopy to monitor mTOR localization. Co-localization coefficient was quantified by co-localization function of image-based software (ZEN) provided by the microscope system. Error bars indicate mean +/− standard error for *n* = 3 independent experiments. Statistical significance was determined via a Student’s *t*-test;* *p* < 0.05. Scale bar: 20 μm.

To elucidate the effect of REP1 on a nutrient signaling pathway, we first tested mTORC1 activity upon REP1 suppression by siRNA treatment. REP1 knockdown in pancreatic cancer cells substantially decreased phosphorylation levels of the downstream targets p70 S6 kinase at T389 (S6K) and S6 ribosomal protein at S235/236 (S6) in spite of similar levels of total p70 S6 kinase seen in Western blotting (Figure 2A). Moreover, REP1 ectopic expression partially restores mTORC1 activity, which is compromised by REP1 knockdown (Figure 2B).

According to recent reports, the subcellular localization of mTOR also reflects the status of mTOR activity, which mostly depends on the intracellular amino acid levels [5]. Under nutrient-complete conditions, the majority of mTOR is localized near lysosomes, whereas mTOR is more likely to be distributed throughout the cytoplasm under nutrient-deficient conditions. The levels of juxta-lysosomal localization of mTOR were assessed by co-localization with alysosomal membrane protein, lysosome-associated membrane glycoprotein 2 (LAMP2), using fluorescence microscopy analysis. As shown in Figure 2C, REP1 knockdown substantially translocated mTOR from a juxta-lysosomal location co-localized with LAMP2 to the cytoplasm under nutrient-complete conditions. The intracellular translocation of *mTOR* to the lysosome can be quantified by calculating the co-localization coefficient between mTOR and LAMP2 using image software provided by confocal microscope systems (Figure 2C), suggesting that REP1 functions to maintain mTORC1 activity in normal growing conditions. These results indicate that REP1 plays a positive regulator in maintaining mTORC1 activity in cancer cells.

### 2.3. REP1 Impacts on Autophagy Activity under Nutrient-Starvation Conditions

**Figure 3:**
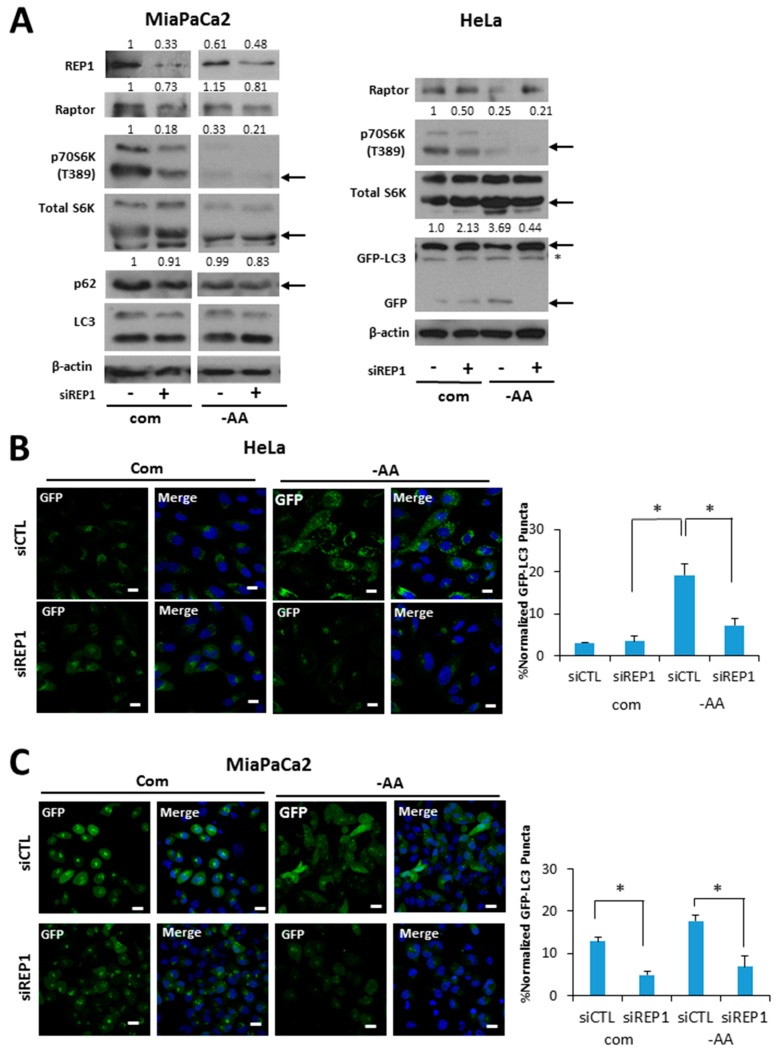

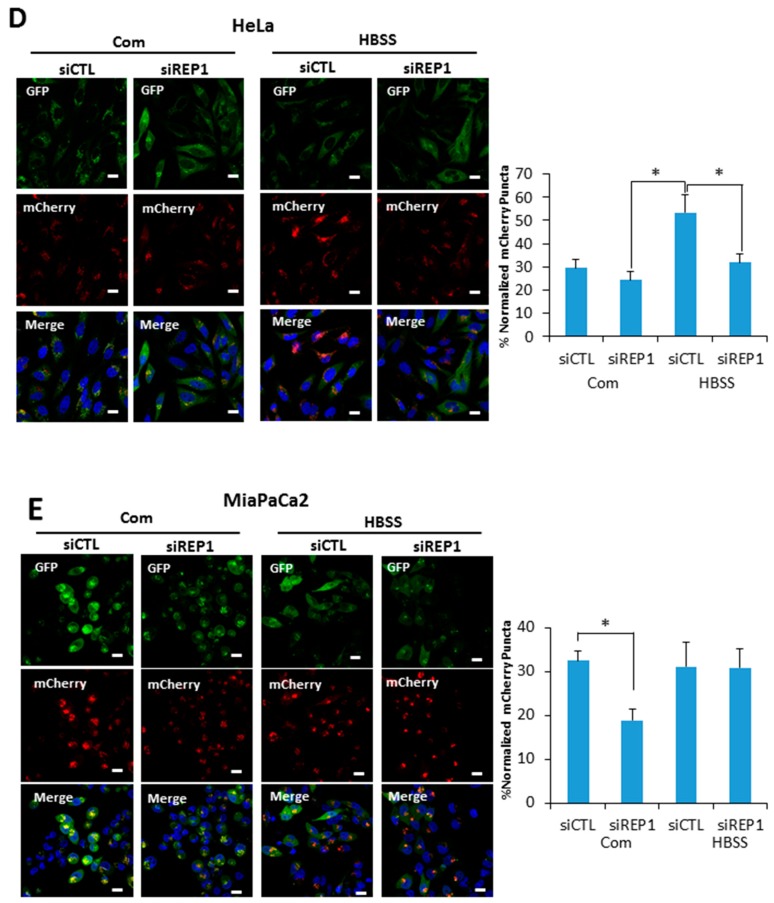
REP1 depletion suppresses starvation-induced autophagy. (**A**) Hela and MiaPaCa2 cells stably expressing GFP-LC3 with a retroviral vector system were treated with CTL or REP1 siRNA and incubated for 48 h. Then, after 2 h of incubation in amino acid-deprived media, cells were harvested and immunoblotted for LC3, P62, and GFP. These results are representative of more than three independent experiments. GFP-LC3 puncta were analyzed by live imaging using fluorescence microcopy in (**B**) Hela and (**C**) MiaPaCa2 cells. Scale bar: 20 μm. Quantification data for GFP-LC3 puncta area are expressed as a percentage of the DAPI area within the cell. Hela and MiaPaCa2 cells stably expressing mCherry-GFP-LC3 with a retroviral vector system were treated with CTL or REP1 siRNA and incubated for 48 h. Then, after 2 h of incubation in Hank’s balanced saline solution (HBSS) media, mCherry puncta from mCherry-GFP-LC3 were imaged by fluorescence microcopy in (**D**) Hela and (**E**) MiaPaCa2 cells. Scale bar: 20 μm. Quantification data for mCherry puncta area derived from mCherry GFP-LC3 are expressed as a percentage of the DAPI area within the cell. Images shown are representative of at least three independent experiments. Statistical significance was determined via *t*-test; * *p* < 0.05.

Autophagy serves as an internal source of nutrients for energy generation during starvation. Thus, it is considered an essential regulator for maintaining cellular metabolic homeostasis. Based on the results that REP1 depletion suppresses mTOR activation, a typical negative regulator of autophagy, we examined the effects of REP1 on autophagy activity. Although the autophagy markers for autophagosome formation and degradation microtubule-associated proteins 1A/1B light chain 3B (hereafter referred to as LC3) and Sequestosome-1(SQSTM1/p62) were not altered by REP1 knockdown (Figure 3A), when cancer cells stably expressing GFP-LC3 were used for autophagy analysis [20], free GFP levels degraded from GFP-LC3 were enhanced under amino acid-deprivation conditions in Western blotting, and were substantially reduced in REP1-depletionconditions (Figure 3A). Next, we monitored autophagy activity in cancer cells stably expressing GFP-LC3 by fluorescence microscopy analysis. As shown in Figure 3B, nutrient deprivation significantly increased the numbers of GFP-LC3 puncta in cells transfected with control siRNA, whereas treatment with REP1siRNA substantially diminished GFP-LC3 puncta formation in both Hela and MiaPaCa2 cells (Figure 3B,C). Interestingly, since pancreatic cancer cells including MiaPaCa2 are reported to harbor relatively high basal activity of autophagy, REP1 knockdown in MiaPaCa2 cells revealed a significant reduction in GFP-LC3 puncta formation even in nutrient-complete media, based on quantification results from autophagosome numbers per cell (Figure 3B). The area levels of green puncta (from GFP-LC3) were quantified by comparison with the total area of cells in theDAPI (4′,6-Diamidino-2-Phenylindole, Dihydrochloride)-stained area (Figure 3B,C). These data suggest that REP1 knockdown inhibits an initiation step of autophagosome formation. Furthermore, we performed autophagy flux analysis with LC3 fused with the tandem fluorophore proteins mCherry-GFP-LC3, to examine whether autolysosome formation can be completed subsequent to initiating autophagosome formation. The mCherry (Red) fluorophore is stable in acidic conditions, while GFP signals are easily quenched. This unique condition allowed us to monitor the status of autophagy process. Initial autophagosome formation occurs in the cytoplasm with neutral pH, which generates fluorescent signals with both green (GFP) and yellow (mCherry and GFP) puncta formation, whereas autolysosomes are formed successfully with lysosomes, resulting in an acidic compartment that confers an increasing number of mCherry red puncta [21]. As shown in Figure 3D, nutrientdeprivation significantly increased the number of red puncta in cells transfected with control siRNA. However, the number of red puncta was significantly decreased in REP1 siRNA-treated cells, indicating that REP1 is more likely involved in autophagosome formation, not in an autolysosome formation step. In the case of pancreatic cells, control siRNA-transfected cells showed high levels of yellow puncta, whereas REP1 depletion with siRNA markedly decreased red puncta in nutrient-complete media, despite the slight increase of GFP puncta from mCherry-GFP LC3 proteins. Moreover, the levels of red puncta from LC3 in response to nutrient starvation are similar in both control and REP1 siRNA-transfected MiaPaCa2 cells, indicating that pancreatic cancer cells undergo metabolic stress, harboring enhanced autophagy activity and showing autophagy dependency, even in nutrient-complete conditions (Figure 3E). Finally, the levels of green puncta (from GFP-LC3) or red puncta (from mCherry-GFP-LC3) were quantified by comparison with the total area of cells in the DAPI-stained area (Figure 3D,E). Taken together, these results indicate that REP1 depletion suppresses relatively high levels of autophagy activity which is induced by either nutrient starvation or oncogenic stress.

### 2.4. REP1 Modulates IntracellularLocalization and Functionof Lysosomes

Our data revealed that REP1 acts as a critical regulator for autophagy, which is particularly activated by nutrient deprivation. Although the number of red puncta from mCherry-GFP-LC3 is markedly decreased in cells transfected with REP1 siRNA, the levels of yellow puncta representing mCherry-GFP-LC3 puncta were not significantly accumulated in REP1 knockdown cells, indicating that REP1 does not appear to be involved in the fusion step between autophagosomes and lysosomes. Moreover, GFP puncta signals derived from mCherry-GFP-LC3 substantially decreased in REP1 siRNA-treated cells, implying that degradation ability against GFP within the lysosome can be impaired by REP1 depletion.

**Figure 4:**
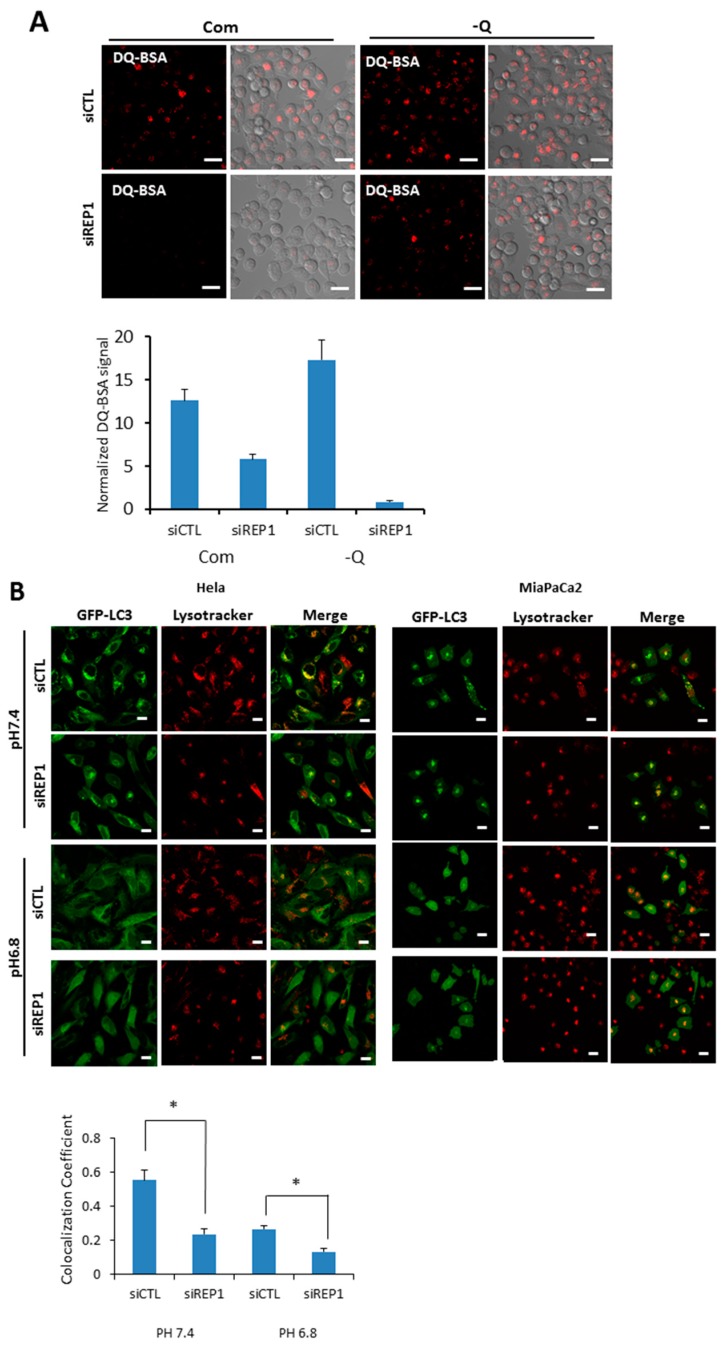
REP1 depletion changes lysosomal function and intracellular localization. (**A**) MiaPaCa2 cells were treated with CTL or REP1 siRNA and incubated for 48 h. Then the cells were treated with DQ-BSA at a final concentration 10 μg/mL for 30 min. Intracellular fluorescent signals were analyzed by fluorescence microcopy and presented as quantification values based on images; (**B**) Hela and MiaPaCa2 cells stably transfected with GFP-LC3 were treated with CTL or REP1 siRNA and incubated for 24 h. Then the cells were further incubated in the media with pH 7.4 or 6.8 overnight and cells were treated with a lysotracker for 30min. Intracellular fluorescent signals were analyzed by fluorescence microcopy and co-localization levels were presented as co-localization coefficient based on image quantification. Error bars indicate mean +/− standard error for *n* = 3 independent experiments. Statistical significance was determined via a Student’s *t*-test; * *p* < 0.05.

To explore the effect of REP1 on lysosome function, we measured lysosomal degradation activity using self-quenched Bodipy dye-conjugated bovine serum albumin (DQ™-BSA), which relies on the cleavage of the self-quenched DQ™ Red BSA protease substrates in an acidic compartment to generate a highly fluorescent fragment. In the results from fluorescence microscopy analysis, pancreatic cancer cells transfected with control siRNA showed red fluorescent puncta in both nutrient-complete and nutrient-deprived media, whereas the levels of red signals de-quenched from DQ-BSA were diminished in the REP1 siRNA-transfected cells. The de-quenching levels showing lysosomal proteolytic activity for DQ-BSA in REP1-depleted cells were significantly decreased compared to the cells transfected with control siRNA, which was more distinct in nutrient starvation conditions (Figure 4A). Next, we monitored the levels of fluorescent puncta stained with lysotrackers, which indicate normal acidic lysosomal conditions. Interestingly, the total area of puncta occupied in cells was not significantly changed by treatment with REP1 siRNA compared to treatment with control siRNA under starvation conditions. These data suggest that lysosomal acidification function does not appear to be impaired significantly by REP1 suppression (Figure S2A). To confirm the effect of REP1 on lysosomal enzyme activity, we directly measured activity and maturation of the lysosomal proteases cathepsin B and D. The protein levels and activity of these enzymes were slightly decreased byREP1 depletion in immunoblot analysis but not by significant levels of changes in REP1 knockdown (Figure S2B). Similarly, the levels of LAMP2, a lysosomal membrane protein, were not significantly changed by REP1 knockdown in the presence or absence of nutrients (Figure S2B).

Recently, it has been reported that cytoplasmic pH can influence lysosome localization and function. Moreover, nutrient deprivation also significantly increases intracellular pH, leading to translocation of lysosomes toward the peripheral membrane of cells [1,22]. To explore the effect of REP1 on cellular pH-mediated lysosomal localization, we first tested intracellular localization of autophagosome in either the presence or absence of REP1. When we examined the co-localization of autophagosomes and lysosomes in REP1 depletion, GFP-LC3 showed a diffuse localization throughout the cytoplasm in nutrient-complete media, but exhibited as puncta forms in cells transfected with control siRNA at pH 7.4 reflecting nutrient starvation, which were mostly co-localized with lysosome stained with the lysotracker. However, the phenotype for lysosomal localization of GFP-LC3 puncta was diminished in REP1 siRNA-transfected cells, which was also similar to the phenotype in the media with either pH 7.4 or 6.8 (Figure 4B). Multiple images from microscope analysis were used for evaluating co-localization coefficient between GFP-LC3 puncta from autophagosome and LysoTracker Red-stained puncta (Figure 4B). Despite the fact that REP1 knockdown lowers the co-localization rate between GFP-LC3 and lysosomes, the intensity of lysotracker-stained puncta in REP1-depleted cancer cells seems to increase based on the image-quantification results. Recent studies reported that lysosomal activity also influencesthe subcellular localization of these organelles, indicating that relatively active lysosomes tend to translocate at peri-nuclear regions. Accordingly, we found that lysosomes stained with LysoTrackers were more likely accumulated near the nucleus in REP1 depletion, which might cause higher levels of lysotrackerpuncta intensity (Figure 4B). These data possibly suggest that REP1 might modulate lysosome-associated proteins for regulation of lysosomal localization and activity within cells.

### 2.5. REP1 Localization Was Affected by Nutrient Status and mTOR Activity

**Figure 5:**
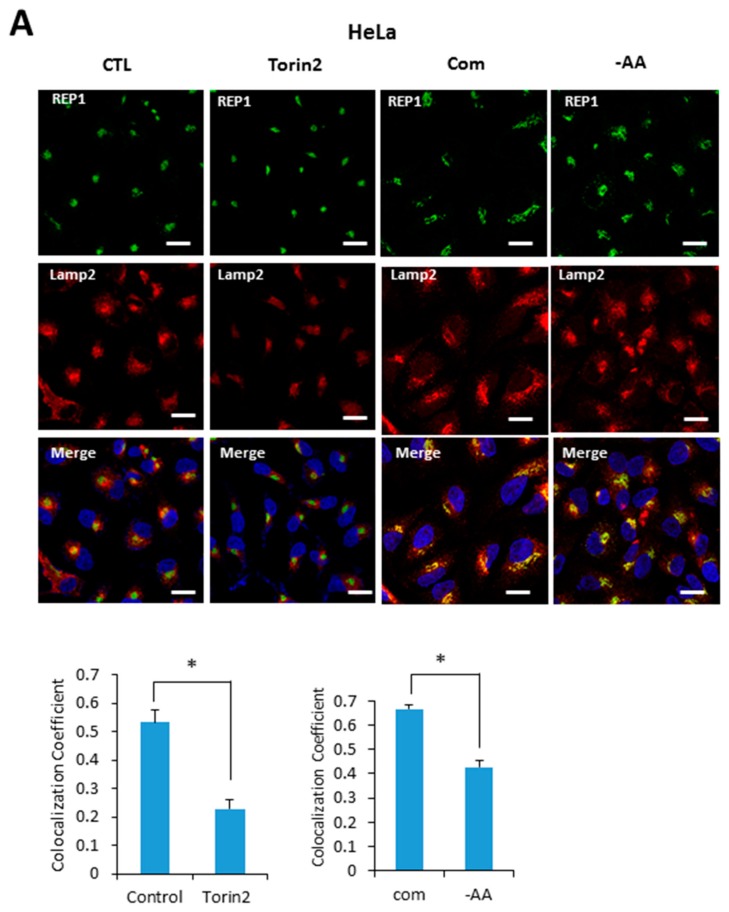

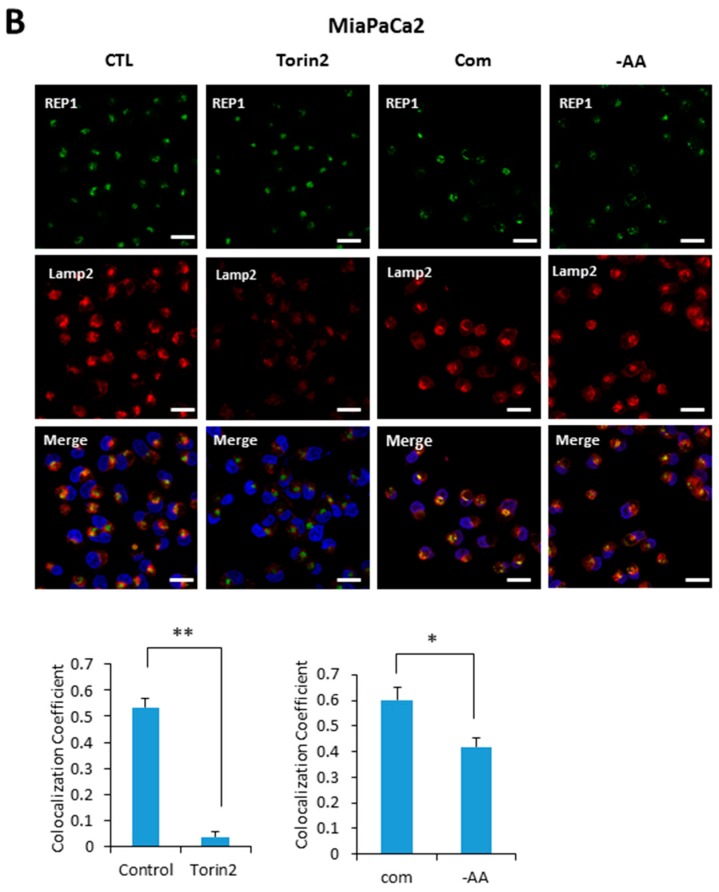
REP1localization is affected by nutrient status and mTOR activity. (**A**) Hela and (**B**) MiaPaCa2 cells were plated and incubated for 24 h, which were further incubated in treatment with Torin2 at 20 nM or replaced with amino acid-deplete media for 4 h. Then the cells were stained with a lysotracker and intracellular fluorescent signals were analyzed by fluorescence microcopy. Co-localization levels were presented as a co-localization coefficient based on image quantification, described in materials and methods. Error bars indicate mean +/− standard error for *n* = 3 independent experiments. Statistical significance was determined via Student’s *t*-test; * *p* < 0.05.

Based on our results indicating that REP1 is required for regulating localization of mTOR and lysosomes, we next examined whether REP1 itself is localized at lysosome, which is affected by mTORC1 activity. Recent studies reported that active mTOR is more likely recruited on the lysosomes, whereas the inactive form of mTOR shows a cytoplasmic diffused pattern by immunofluorescence analysis. Indeed, REP1 protein was distributed over cytoplasm as irregular small puncta forms which were partially co-localized with LAMP2, a lysosomal marker under nutrient-complete conditions. Interestingly, upon treatment with Torin2, an mTOR inhibitor, REP1 was more likely accumulated near the peri-nuclear region, which abolished its co-localization with LAMP2 in both Hela (Figure 5A) and MiaPaCa2 cells (Figure 5B). The co-localization levels of REP1 and LAMP2 can be quantified by calculating the co-localization coefficient using image software (Figure 5A,B). Similar to the phenotype in mTOR inhibitions, the co-localization levels of REP1 and LAMP2 were also reduced in response to amino acid deprivation (Figure 5A,B). These results suggest that intracellular localization of REP1 could be regulated by mTOR activity, implying that interrelation between REP1 and mTOR is critical to maintaining functional vesicle trafficking, including regulation of autophagosomes and lysosomes.

### 2.6. REP1 Regulates Macropinocytosis

**Figure 6:**
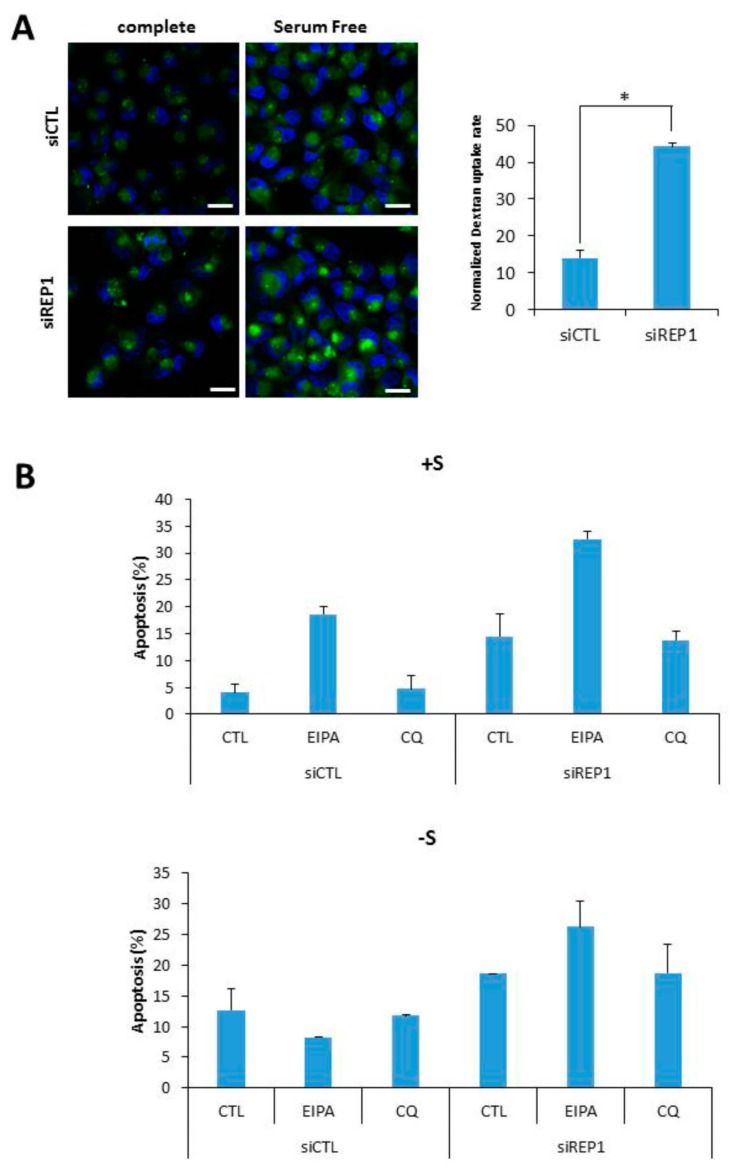
REP1 depletion induces macro pinocytosis (**A**) MiaPaCa2 cells were treated with CTL or REP1 siRNA for 24 h and replaced with serum deplete media to incubate further for another 12 h. Then the cells were treated with FITC-dextran at final concentration of 10 µg/mL for 30min and analyzed for cellular uptake of FITC-dextran by fluorescence microscopy. The level of macropinocytic uptake was quantified by image-based determination of the total macro pinocytic vesicle area compared with DAPI-stained area within cell. Data are expressed as a percentage of the DAPI-stained area within the cell area Statistical significance was determined via *t*-test; * *p* < 0.05; (**B**) MiaPaCa2 cells were transfected with CTL or REP1 siRNA for 24 h and replaced with either serum complete- or deplete-media for further incubation. Concomitantly with media replacement, either EIPA or Chloroquine (CQ) was treated respectively at final concentration of 15 μM in either REP1 or CTL siRNA. After 48 h of further treatment, cell death assay was performed using Annexin V/PI staining to show the cell apoptotic rate. Error bars indicate mean +/− standard error for *n* = 3 independent experiments.

Recently, we and others have suggested that a type of endocytosis, macro pinocytosis can be utilized to support cell growth and survival under nutrient-starvation conditions, throughout cellular uptake and degradation of extracellular macromolecules including proteins like bovine serum albumin (BSA) [9,23]. In this study, we examined whether REP1directly regulates macropinocytosis using a marker molecule, FITC-Dextran. To investigate the effect of REP1 on macropinocytosis, we performed fluorescence microscopy analysis to monitor the intracellular uptake of dextran as a marker of macropinocytosis in the presence or absence of REP1.Unexpectedly, REP1-depleted cells revealed substantially increasing levels of macro pinocytosis in MiaPaCa2 pancreatic cancer cells. As shown in Figure 6A, macro pinosomes harboring FITC-dextran were accumulated at higher levels of puncta in REP1 knockdown cells compared to in control siRNA-transfected cells. The total area of green puncta taken-up from FITC-dextran was quantified by comparison with the total area of cells in the DAPI-stained area (Figure 6A). In addition, these macropinosome-vesicles were mostly localized at peri-nuclear regions of REP1-depleted cells, suggesting that REP1 also modulates macropinosome trafficking which might be closely associated with subcellular localization of lysosomes (Figure 6A). Next, to determine the effect of REP1-mediated macropinocytosis and lysosomal movement on cancer cell survival, we performed apoptosis assay using Annexin V/PI after treatment with either multiple macro pinocytosis/autophagy regulators or REP1siRNA, or both. The increase in macropinocytosis upon glutamine deprivation was significantly inhibited by 5-(*N*-ethyl-*N*-isopropyl)-amiloride (EIPA), which is known as a macropinocytosis inhibitor [9,23]. Indeed, either suppression of REP1 or macropinocytosis inhibition alone had a relatively low effect on apoptotic cell death, whereas cell death rate was significantly induced upon both suppression of REP1 and macropinocytosis. In comparison, chloroquine (CQ), a lysosomotropic agent known as one of autophagy inhibitors, showed a relatively weak effect on cell death in combinatorial treatment with siREP1 (Figure 6B). These data indicate that REP1 plays a critical role in cancer growth and survival in response to nutrient deprivation via multiple mechanisms including macropinocytosis, in addition to autophagy and lysosome functional regulation.

## 3. Discussion

REP1 has been characterized as a crucial molecule for prenylation of the Rab protein and its mutation is tightly associated with developmental diseases as a result of a wide variety of types of tissue degeneration, including retinal and choroidal degeneration during eye development [14,15] and degeneration in brain development in the zebrafish model [17]. In addition to tissue-degenerative phenotypes, knockout of the REP1 gene in zebrafish exhibited early embryonic lethality [16]. Recent reports have demonstrated that REP1 is closely linked to cell proliferation and survival in the neoplastic stage through regulating translocation for either a growth factor receptor or a transcription factor to prevent cell death. Indeed, REP1 plays an important role in cancer progression, which is regulated by intracellular localization of EGFR or FOXO3 [18,19].

Since REP1 is involved in Rab protein recruitment in proper vesicles during vesicle trafficking, and the REP1mutation induces cell death which might cause tissue degeneration, we hypothesized that REP1 plays a critical role in the regulation of autophagy, which requires a variety of RabGTPases at distinct steps of vesicle trafficking processes [12]. In addition, overall cellular phenotypes of Rep1 depletion or mutation are similar to that of those seen in autophagy defects. Moreover, a number of Rab proteins are known to be involved in distinct steps of the autophagy pathway, which is a lysosome-mediated degradation process and is induced by typical metabolic stresses including growth factor- or nutrient-deprivation and accumulation of reactive oxygen species (ROS) [24]. Rab proteins that influence autophagy—such as Rab1, Rab5, Rab7, and Rab11—can be target proteins for lipid modification regulated by REP1 [25]. Since nutrient deprivation eliminates mTOR activity completely, we hypothesized that REP1 work sin a distinct mechanism to maintain cell growth and survival in response to nutrient deprivation.

According to the data that REP1knockdown sensitizes cancer cells, especially under nutrient-deprivation conditions, we hypothesized that target of rapamycin (TOR) signaling can be closely related to REP1-mediated cell growth and survival to sustain metabolic stress conditions. TOR signaling is conserved as a nutrient-sensing protein from yeast to humans which controls cell growth and proliferation via regulating ribosome biogenesis, protein translation, and even autophagy. Furthermore, a yeast homolog of the vertebrate *choroideremia* (*chm*, *REP1*) gene, named *MRS6*, has a role in regulating TOR signaling and ribosome biogenesis, which is dependent on extracellular nutrient levels in the yeast model system [26,27], implying that REP1 has a conserved role in association with the nutrient signaling pathway, TOR.

Based on our data, there is a relationship between REP1 and autophagy which is mediated by the regulation of mTORC1 signaling and lysosomes. REP1 knockdown suppressed mTORC1 activity, a negative modulator of autophagy in normal growing conditions. During nutrient starvation, REP1 depletion clearly blocked autophagy stimulated by various metabolic stresses, thereby exhibiting severe suppression of cell proliferation and survival in REP1 knockdown cells in response to metabolic stress, commonly shown in cancer development.

Although this detailed mechanism needs to be defined further, our results suggested that REP1 might modulate intracellular localization of the lysosome as well as autophagy pathway during starvation, which is affected by mTOR activity. Moreover, normal recruitment of REP1 itself to the lysosomes is significantly impaired in mTOR inactivation or nutrient deprivation, including deprivation of amino acids and growth factors. Unexpectedly, REP1 knockdown increases macropinocytosis, a nonselective endocytosis process for utilization of extracellular macromolecules. However this phenotype—increased level of uptake of macropinocytic marker—might be a feedback phenotype to compensate autophagy suppression shown in REP1-depleted cells.

Autophagy is generally necessary for modulating metabolic disturbances caused by a variety of stresses, including lack of nutrient or oxygen levels and oxidative stress. Dysregulation of autophagy can be closely linked to the progression of human diseases, including cancer, neurodegenerative disorders, and metabolic diseases. Therefore, understanding the regulatory mechanisms of macromolecule degradation pathways including autophagy can assist us in exerting therapeutic effects for several disorders. Particularly, autophagy plays context-dependent roles in tumor progression, relying on developmental stages or the microenvironment of tumor [4].

In summary, we proposed a novel mechanism in which REP1 plays a critical role in cancer cell growth and survival mediated through regulating mTORC1 activity. As a downstream effector of mTORC1, lysosomal degradation pathways including autophagy and macropinocytosis can be modulated by REP1, which directly influences intracellular localization of mTOR and lysosomes to determine their activities. Therefore, understanding the molecular mechanism for inter-regulation among REP1, mTORC1, and lysosomal degradation pathways would provide an intriguing rationale for targeting for pancreatic cancers with high capacities for lysosomal degradation. Taken together, our study suggested that the molecular mechanisms of REP1 in regulating mTOR and subsequent lysosomal degradation pathways, as well as targeting autophagy, might be possible therapeutic options for cancer patients with high expression of REP1 levels.

## 4. Materials and Methods

### 4.1. Chemicals and Reagents

Primary antibodies against phospho-S6 (S235/236), S6 ribosomal protein, phospho-p70S6K (T389), p70S6K, and LC3B, respectively were purchased from Cell Signaling Technology (Beverly, MA, USA). Primary antibodies were purchased against LAMP2 from Abcam (Cambridge, UK), P62 from Progen (Heidelberg, Germany), β-actin from Sigma-Aldrich (St. Louis, MO, USA) and tubulin, cathepsin B, and cathepsin D from Santa Cruz (Dallas, TX, USA). The secondary antibodies, Alexa Fluor-488 conjugated anti-rabbit (A21206) and Alexa Fluor-592 anti-mouse (A11005), were from Life Technologies (Carlsbad, CA, USA). Horseradish peroxidase (HRP)-linked anti-rabbit and anti-mouse antibodies were from Bethyl Laboratories (Montgomery, TX, USA). DAPI, dextran, fluorescein (70 KDa; D1820), and dextran, tetramethylrhodamine (70 K Da; D1817) were from Life Technologies (Carlsbad, CA, USA). Mounting media (S3023) was from DAKO (Carpinteria, CA, USA). Bovine serum albumin (BSA; A1470), chloroquine (CQ; C6628), 5-(*N*-Ethyl-*N*-isopropyl), amiloride (EIPA; A3085), and the phosphatase inhibitor cocktail were from Sigma-Aldrich (St. Louis, MO, USA). The protease inhibitor cocktail tablet was from Roche Applied Bioscience, Penzberg, Upper Bavaria, Germany Rapamycin (S1039) and Torin2 were purchased from Selleck Chemicals (Houston, TX, USA).

Non-essential amino acids (1567906), essential amino acids (1542383), glutamine (25030081), HEPES (1627660), vitamin solution (1567870), and sodium bicarbonate (1546264) were from Life Technologies (Carlsbad, CA, USA). Mounting media (S3023) was from DAKO (Carpinteria, CA, USA).

### 4.2. Cell Lines and Culture Conditions

The Hela cells, and Panc1, 8988T, and MiaPaCa2 human pancreatic cancer cell lines from the American Type Culture Collection (ATCC; Manassas, VA, USA) were kindly provided by Kyung-Tae Kim and Yun-Hee Kim (National Cancer Center, Goyang-si, Korea). All cells were maintained under 5% CO_2_ at 37 °C in medium supplemented with 10% fetal bovine serum (FBS) (Hyclone), 100 U/mL penicillin, and 100 μg/mL streptomycin (Life Technologies). Mouse embryonic fibroblast (MEFs) and MiaPaCa2 were maintained in Dulbecco’s modified Eagle’s medium (DMEM; Life technologies). For glutamine starvation, DMEM without glutamine (Life Technologies) was supplemented with 10% dialyzed FBS (Life Technologies). For the amino acid-starvation medium, Hank’s balanced saline solution (HBSS) was supplemented with 10% dialyzed FBS, glucose, vitamins, HEPES and minerals at the same concentrations as in DMEM.

GFP-LC3 in the MigRI-based retroviral vector was generously provided by Craig Thompson andpBabemCherry-GFP-LC3 (#22418) was a gift from Jayanta Debnarth through Addgene [28]. Hela and MiaPaCa2 stably expressing GFP-LC3or mCherry-GFP-LC3 were generated following standard protocols for retrovirus transduction. The sequences of siRNA for REP1 are 5′-CCGGAGAGUUCUGCAUGUU-3′ (#1) and 5′GCAUGAAAGGCACCUAUUU-3′ (#2).

### 4.3. Western Blotting

For Western blotting, cells were rinsed with ice-cold phosphate buffered saline (PBS) and harvested using ice-cold lysis buffer (RIPA buffer). Samples were then incubated in RIPA buffer (50 mM TRIS-Cl pH 7.4, 150 mMNaCl, 1% NP-40, 0.5% Na-deoxycholate, 0.1% Sodium dodecyl sulfate, (SDS), and 1 mM ethylenediaminetetraacetic acid (EDTA) with protease inhibitor cocktail (Roche Applied Bioscience, Penzberg, Upper Bavaria, Germany) and phosphatase inhibitor (Sigma-Aldrich, St. Louis, MI, USA) for 15 min, and soluble lysate fractions were isolated by centrifugation at 16,000× *g* for 15 min. Protein concentrations were determined with the Pierce BCA Protein Assay (Thermo Scientific, Waltham, MA, USA), and equal amounts of protein were analyzed by SDS gel electrophoresis and Western blotting following standard protocols.

### 4.4. siRNA and Transfection

REP1 siRNA and negative control siRNA (non-targeting pool) were purchased from Genolution Inc. (Seoul, Korea); cells were transfected with lipofectamine 2000 for siRNA in Opti-MEM media. Cells were then resuspended in complete DMEM medium, incubated for 24–48 h, and used for further experiments.

### 4.5. Immunofluorescence Analysis

To determine the localization of mTOR, immunostaining against mTOR and LAMP2 was performed using MiaPaCa2 and Hela cells. After they were plated onto glass coverslips in complete medium for 18 h, cells were incubated in control (CTL) and REP1 siRNA treatment for 24 h. For immunostaining, cells were rinsed with ice-cold PBS, fixed with 3.7% formaldehyde for 30 min, and permeabilized with 0.05% Triton X-100 in PBS (PBS-T) for 5 min. After rinsing with PBS-T, cells were blocked with blocking buffer (0.5% BSA in PBS-T) for 1 h, incubated with primary antibodies in blocking buffer for 16 h in 4 °C, washed three times with PBS-T and then incubated with secondary antibodies conjugated with fluorescent probe in PBS-T for 1 h at room temperature. After three PBS-T washes, cells were stained with DAPI for 10 min and mounted on microscope slides. A confocal fluorescence microscope (Zeiss LSM 780, Oberkochen, Germany) was used for imaging and analysis. Microscopic images were generated through capturing at least five different areas per culture condition from the cells with either 400× or 630× magnification. The experiments were repeated at three independent days. Images were quantified by either counting function of pixel area or co-localization function ofimage-based software (ZEN black edition), which is provided by the confocal microscope system (LSM780).

Pearson’s correlation coefficient was used for calculating co-localization.

$$R_{P}=\frac{\sum_{i}\left(ch1_{i}-ch1_{aver}\right)\times \left(ch2_{i}-ch2_{aver}\right)}{\sqrt{\sum_{i}{\left(ch1_{i}-ch1_{aver}\right)}^{2}\times {\left(ch2_{i}-ch2_{aver}\right)}^{2}}}$$

Pearson’s correlation coefficient provides information on the intensity distribution within the co-localizing region. Actual value ranges from−1 to +1. 0 mean pixels in the scattergram distribute in a cloud with no preferential direction. −1 and +1 mean all pixels are found on straight line in the scattergram.

### 4.6. Lysosome Trafficking Assay

For imaging of lysosome trafficking mediated by extracellular pH, Hela, and MiaPaCacells stably expressing mCherry-GFP-LC3were transfected with scrambled siRNA(CTL) or REP1 siRNA following the reverse transfection protocol, and incubated at 37 °C for 24 h in an 8-well Lab-Tek chamber. Subsequently, cells were changed with pH7.4 and pH6.8 DMEM media for 16 h. Then, nuclei were stained using DAPI. For lysotracker staining, LysoTracker Red was treated on the cells 30 min prior to imaging. Images were captured using an LSM780 fluorescent microscope (Zeiss, Oberkochen, Germany) and quantified using the software ZEN (Zeiss, Oberkochen, Germany). Auto lysosome signals (Red) from mCherry-GFP-LC3 were captured in at least five distinct fields from different regions for an individual experimental set. The total puncta area per cell was normalized by the area of DAPI-stained nucleus of that cell.

### 4.7. Macropinocytosis Analysis

For imaging of macropinocytosis, cells were incubated in the media without FBS for 4 h to increase macro pinocytosis. For starvation, the medium was replaced with either serum- or glutamine-complete or serum- or glutamine-deplete medium. These cells were incubated with 0.5 mg/mL dextran conjugated with FITC for the indicated periods at 37 °C. Subsequently, cells were washed three times with ice-cold PBS and fixed with 3.7% formaldehyde in PBS for 15 min. Nuclei were then stained using DAPI and mounted using mounting media (DAKO). Images were captured using an LSM780 confocal fluorescent microscope (Zeiss, Oberkochen, Germany) and quantified using software ZEN (Zeiss, Oberkochen, Germany). The total macropinosome area per cell was normalized by the area of DAPI-stained nucleus of that cell. Macropinosome areas were quantified in at least five distinct fields captured from different regions for an individual experimental set. In addition, subcellular localization of GFP-LC3 or mCherry-GFP-LC3 for measuring autophagy activity was monitored by LSM780 confocal fluorescent microscope (Zeiss, Oberkochen, Germany).

### 4.8. Cell Growth and Viability

Cells were plated in complete media in 24-well plates prior to starvation. Twenty-four hours after seeding, cells were washed with PBS and incubated in the indicated glutamine- or serum-starvation media with 10% dialyzed FBS. For rescue experiments, the cells were incubated in the media supplemented with either 0% BSA or 4% BSA. Negative control cells for macropinocytosiswere treated with EIPA. The number of viable cells was assessed using automated cell proliferation detector using IncuCyte^TM^ (Essen Instruments, Ann Arbor, MI, USA). Proliferation was measured through quantitative kinetic processing metrics derived from time-lapse image acquisition and presented as percentage of culture confluence over time. The number of cells wasalso counted either using a Coulter Counter (Beckman Coulter, Brea, CA, USA) and/or using trypan blue exclusion and an automated cell counter (Nano-Entek, Seoul, Korea). Cell viability was determined by Annexin V and propidium iodide (PI) staining following standard protocols at indicated periods of time (BD Biosciences). Cells negative for both Annexin V and PI staining were considered live cells.

## Supplementary Materials

Supplementary materials can be found at www.mdpi.com/1422-0067/18/9/1866/s1.
