# Supplementary material for: REP1 Modulates Autophagy and Macropinocytosis to Enhance Cancer Cell Survival
Source: Int J Mol Sci. 2017 Aug 28;18(9):1866. doi: 10.3390/ijms18091866 (PMC5618515; doi:10.3390/ijms18091866)
Supplement: Supplementary file 1 [file ijms-18-01866-s001.pdf]

Supplementary Materials: REP1 Modulates Autophagy and Macropinocytosis to Enhance Cancer Cell Survival

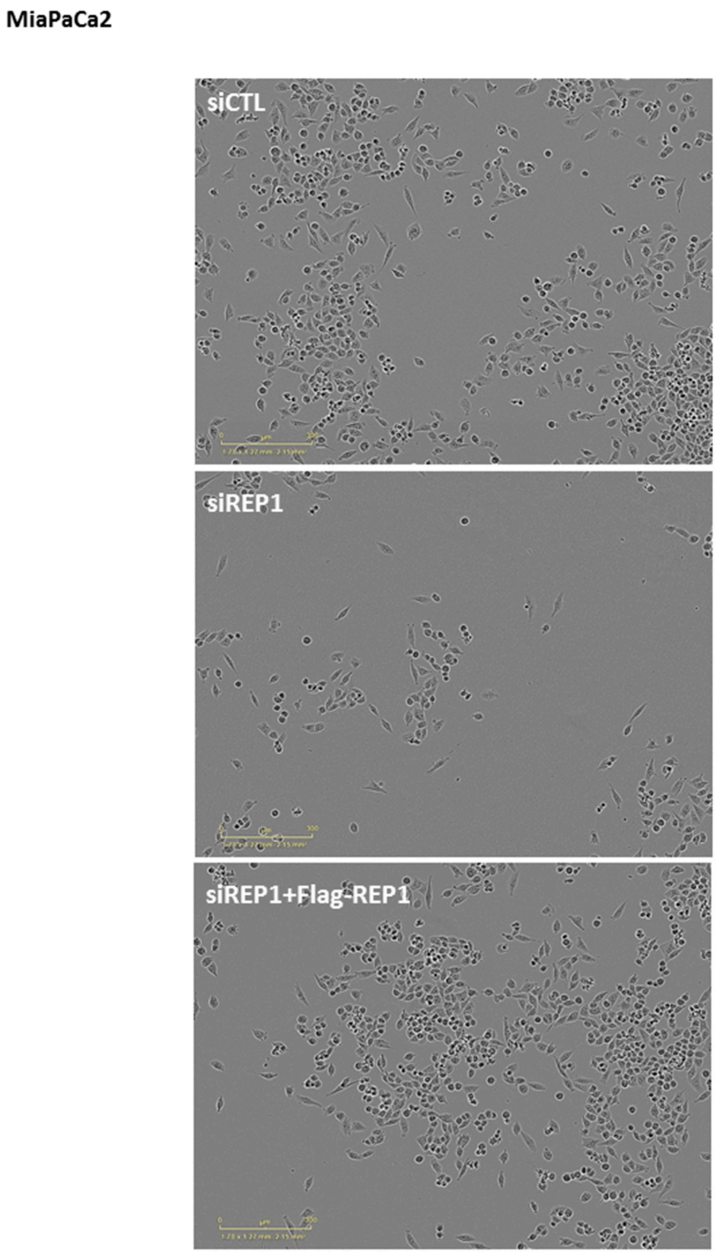

Figure S1. Bright-field cell images were taken at 60 h incubation time point in the IncuCyte™ for monitoring cell proliferation. MiaPaCa2 cells were treated with CTL and REP1 siRNAs 24 hr prior to transfecting Flag-REP1 plasmid onto REP1 siRNA treated cells. Then these cells were incubated in the IncuCyte™ for measuring cell proliferation. Scale bar: 300 μm (Figure 1A).

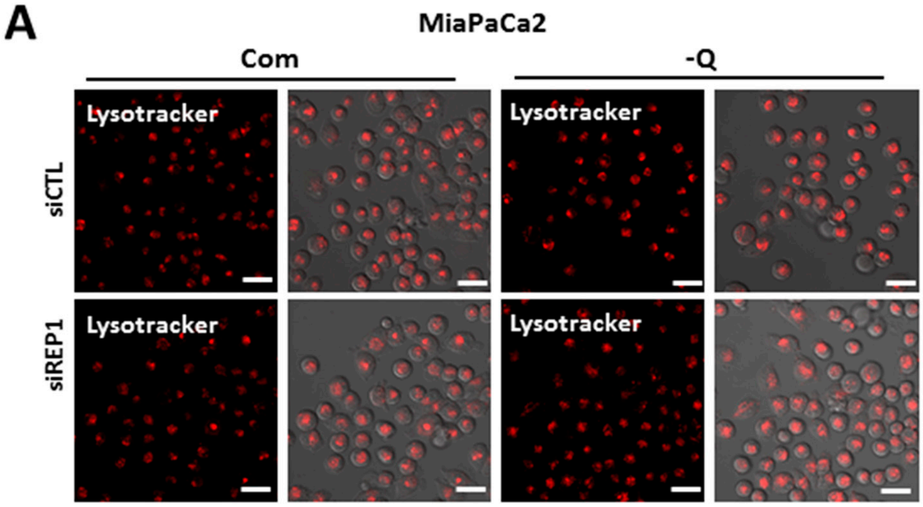

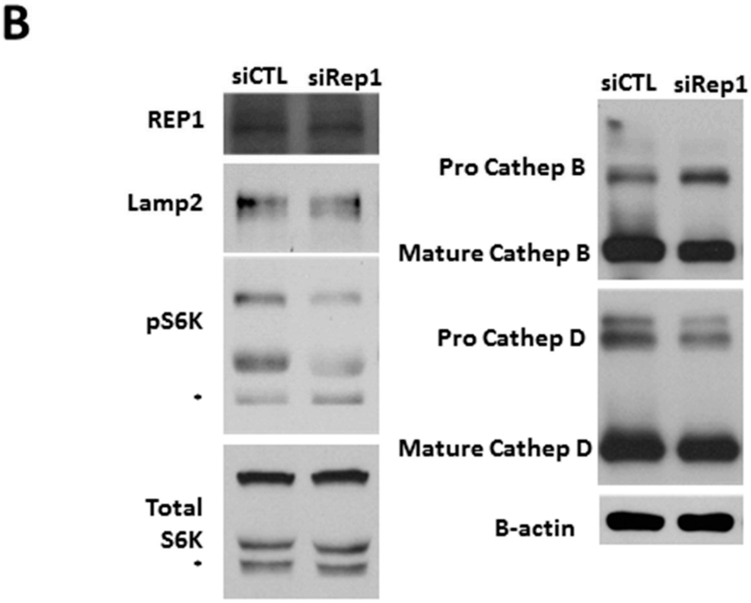

Figure S2. Lysotracker (A) at 100 nM for 30 min which was replaced with glutamine-free medium and incubated for another 4 h. Intracellular fluorescent signals were analyzed by fluorescence microcopy. (B) Hela and MiaPaCa2 cells were treated with CTL or REP1 siRNA and incubated for 48 h. Then, cells were harvested and the lysates were immunoblotted with antibodies against REP1, Lamp2, p70S6K, total 70S6K, Cathepsin B and D, and beta actin.
